# Subjective memory complaints and cognitive performance in a sample of healthy elderly

**DOI:** 10.1590/S1980-57642009DN20100009

**Published:** 2008

**Authors:** Paulo Caramelli, Rogério Gomes Beato

**Affiliations:** Behavioral and Cognitive Neurology Unit, Department of Internal Medicine, Faculty of Medicine, Federal University of Minas Gerais, Belo Horizonte, Brazil.

**Keywords:** aging, memory, cognition, neuropsychological tests, envelhecimento, memória, cognição, testes neuropsicológicos

## Abstract

Memory loss is a major complaint among the elderly population. However, the clinical significance of this symptom is variable and also controversial in the scientific literature.

**Objective:**

To compare the cognitive performance of two groups of healthy elderly, one group with and the other without, subjective memory complaints (SMC).

**Methods:**

Sixty cognitively intact elderly individuals (39 females and 21 males), aged 69.9±6.3 years and with educational level of 8.5±5.5 years, were included in the study. Participants were submitted to the Mini-Mental State Examination and to the Cornell depression scale in order to rule out global cognitive impairment and depression, respectively. Moreover, they answered the MAC-Q, a questionnaire devised to evaluate subjective impression of memory function. Subsequently, they were submitted to the digit span forward and backward, the Brief Cognitive Screening Battery, and to the Frontal Assessment Battery.

**Results:**

Twenty-seven individuals had MAC-Q scores <25 and thus were classified as not having SMC, while 33 had MAC-Q scores ≥25 and were considered to have SMC. No differences for age, gender, education and MMSE scores were found between the two groups. The comparison between the performance of the groups of complainers and non-complainers on the different cognitive tests yielded no significant difference, although there was a trend toward non-complainers performing better on incidental memory.

**Conclusions:**

The presence of SMC was not associated to objective memory impairment or to other cognitive deficits in this group of elderly individuals.

## Abstract (pt)

Perda de memória é uma queixa freqüente na população idosa. Entretanto, seu significado clínico é variável e controverso na literatura científica.

**Objetivo:**

Comparar o desempenho cognitivo de dois grupos de idosos saudáveis, um com e outro sem queixas subjetivas de memória.

**Métodos:**

Sessenta idosos sem comprometimento cognitivo (39 mulheres e 21 homens), com idade de 69,9±6,3 anos e com escolaridade de 8,5±5,5 anos, foram incluídos no estudo. Todos foram submetidos ao Mini-Exame do Estado Mental (MEEM) e à Escala de Depressão de Cornell para exclusão, respectivamente, de comprometimento cognitivo global e de depressão. Também responderam ao MAC-Q, questionário elaborado para avaliar a impressão subjetiva do funcionamento da memória. Posteriormente, eles foram submetidos aos testes extensão de dígitos em ordem direta e inversa, à Bateria Breve de Rastreio Cognitivo e à Bateria de Avaliação Frontal.

**Resultados:**

Vinte sete idosos tiveram escores <25 no MAC-Q e foram, portanto, classificados como não tendo queixas, enquanto 33 tiveram >25 pontos no questionário e foram considerados como tendo queixas. Não houve diferenças entre os dois grupos em relação à idade, gênero, escolaridade e pontuações no MEEM. A comparação entre o desempenho dos idosos com e sem queixas nos diferentes testes não revelou diferenças significativas, embora tenha sido observada tendência para que os sem queixas tivessem melhor desempenho de memória incidental.

**Conclusões:**

A presença de queixas subjetivas de memória não esteve associada a déficits objetivos de memória ou de outras funções cognitivas neste grupo de idosos saudáveis.

Memory loss is one of the most common complaints arising in consultations with elderly people, being reported by 25% to 50% of these individuals.^1^ However, whether these subjective memory complaints (SMC) are related to objective memory deficits or to subsequent development of dementia, remains a matter of debate.

A recent review found that SMCs are not consistently associated with current cognitive impairment, but rather are associated with a greater risk of future cognitive decline.^2^ Indeed, the diagnosis of mild cognitive impairment (MCI), which entails an increased likelihood of conversion to dementia, demands the existence of SMCs, preferably confirmed by an informant.^3^

High age, female gender and low educational level are generally associated with a higher prevalence of memory complaints.^1^ In an autopsy study, SMCs were found to be related to the presence of Alzheimer’s disease (AD) pathology in elderly with and without dementia, suggesting that memory complaints in older persons may be a sign of self awareness of a degenerative process.^4^

However, SMCs might also be related to depression and some personality traits, such as neuroticism.^2^ It is also possible that these complaints vary according to the culture of the people studied. In a recent Brazilian study, Minett et al. found that subjects with and without SMCs performed similarly in a series of cognitive tests, although the former had higher scores on the Geriatric Depression Scale.^5^

The present study aimed to further investigate this topic in a group of cognitively healthy Brazilian elderly subjects which were divided into two subgroups according to the presence of SMCs and submitted to brief cognitive tests.

## Methods

Sixty cognitively intact elderly individuals (39 females and 21 males), aged 69.9±6.3 years (ranging from 60 to 91 years), and with mean educational level of 8.5±5.5 years (ranging from 1 to 20 years), were included in the study. These individuals were family caregivers of demented patients followed at the Behavioral and Cognitive Neurology Unit of the Faculty of Medicine of the Federal University of Minas Gerais, in Belo Horizonte (MG), Brazil, and also volunteers recruited from the community.

Inclusion criteria were absence of neurological or psychiatric diseases according to a clinical interview, absence of depression (see below), and no use of benzodiazepines, antidepressants or neuroleptics.

All participants were submitted to the Mini-Mental State-Examination (MMSE)^6,7^ and to the Cornell scale of depression.^8,9^ Performance on the MMSE was adjusted for educational level and had to be greater than or equal to 21 for 1-3 years of schooling, greater than or equal to 24 for 4-7 years and greater than or equal to 26 for individuals with 8 or more years of schooling.^10^ Scores on the Cornell scale of depression had to be less than or equal to 7 points in order to rule out depression.^8^

Cognitive evaluation was carried out with the following tests: the Brief Cognitive Screening Battery (BCSB)^11,12^, digit span forward and backward and the Frontal Assessment Battery (FAB).^13,14^ The BCSB includes a memory test of 10 simple figures and yields different scores, namely: incidental and immediate memory, learning, delayed recall and recognition.^15,16^ The battery also includes a category fluency test (animals per minute) and clock drawing and has proven very sensitive in the diagnosis of mild AD.^12^ The FAB is a brief diagnostic instrument for the assessment of executive functions in patients with suspected frontal lobe syndrome.^13^

All individuals were given a structured self-report memory questionnaire, the MAC-Q.^17^ This questionnaire was devised to assess age-related memory decline. It is composed by six questions related to memory functioning in everyday situations (e.g., to remember a telephone number that he/she uses at least once a week) in which the subject is asked to compare and rate his/her current ability to when he/she was 40 years’ old. The total score on the MAC-Q ranges from 7 to 35, where greater scores indicate subjective memory loss. Scores greater than or equal to 25 have been found to be suggestive of age-associated memory impairment. Accordingly, in the present study, the individuals were divided into two groups: absence of SMCs (MAC-Q scores <25) and presence of SMCs (MAC-Q scores ≥25). The performance of the two groups on the different cognitive tests was compared.

One of the authors administered the MMSE, the Cornell scale and the MAC-Q. Subsequently, the other investigator, blinded to the subjects’ results for these three measures, administered the cognitive evaluation.

Descriptive analysis of the data and statistical comparisons between the performances of the two groups on the different cognitive tests were carried out with MedCalc software. Student’s t-test was used for comparison of age, educational level and MMSE scores, as well as for the results of the other cognitive tests (digit span, BCSB and FAB). Chi-square was employed for comparing gender distribution of the two groups. Level of significance was set at 0.05.

The study was approved by the Research Ethics Committee of the Federal University of Minas Gerais and all participants signed the approved written informed consent.

## Results

Twenty-seven individuals had MAC-Q scores <25 and thus were classified as non-complainers. These were 14 women and 13 men, aged 69.6±5.4 years, with mean educational level of 8.8 years. Mean MMSE score of this group was 27.8.

The group of complainers (MAC-Q scores =25) was composed by 25 women and 8 men, aged 70.3±7.0 years, with mean educational level of 8.2 years. Mean MMSE score of this group was 27.2.

No significant difference for age, gender, education and MMSE scores were found between the two groups. Table 1 depicts the main demographic data as well as the MMSE and MAC-Q values for both groups.

**Table 1:** Demographic data, MMSE and MAC-Q scores from the group of non-complainers and complainers.

Variable	Non-complainers	Complainers	p
N	27	33	
Age*	69.6±5.4	70.3±7.0	0.66
Gender	14F / 13 M	25F / 8 M	0.09
Educational level*	8.8±5.5	8.2±5.6	0.68
MMSE*	27.8±1.5	27.2±1.8	0.23
MAC-Q scores*	20.9±3.1	28.3±2.8	-

N: number of individuals; MMSE: Mini-Mental State Examination; MAC-Q: Memory Complaint Questionnaire.

* Results represent mean values±standard deviation.

The comparison between the performance of the groups of complainers and non-complainers on the different cognitive tests yielded no significant difference, although there was a trend for non-complainers to perform better in incidental memory.

Table 2 presents the scores from the two groups in all tests as well as the statistical comparisons between them.

**Table 2:** Comparison between the groups of complainers and non-complainers in the different cognitive tests.

Cognitive test	Non-complainers	Complainers	p
Digit span forward	5.1±1.1	4.9±1.0	0.44
Digit span backward	3.7±1.0	3.4±0.9	0.26
Incidental memory	6.2±1.4	5.5±1.2	0.06
Immediate memory	8.1±1.4	7.9±1.2	0.57
Learning	8.6±1.2	8.9±1.1	0.35
Delayed recall	8.3±1.4	8.1±1.3	0.63
Recognition	9.9±0.3	9.8±0.4	0.69
Category fluency	17.8±5.1	16.0±4.1	0.13
Clock drawing	8.1±1.9	7.8±1.9	0.57
FAB total score	13.3±2.4	12.9±2.4	0.54

FAB: Frontal Assessment Battery. Results represent mean values±standard deviation.

## Discussion

In the present study, we found no significant difference in the performance of elderly subjects with and without SMCs on a series of brief cognitive tests assessing attention, episodic and semantic memory, and executive functions. Only a trend toward a significant difference emerged for the subtest of incidental memory from the BCSB, an item that is more related to attention than to memory itself.

Some previous studies have found a relationship between SMCs and objective memory performance, while many others have not.^2^ The methodology adopted by the different studies in assessing SMC varies, where some use only a simple question about perceived memory problems, while others base this classification on the results of specific questionnaires, such as the MAC-Q used in our study.

Interestingly, a previous Brazilian study compared the use of the MAC-Q with direct questioning about memory problems, and found that a significant percentage of the sample had SMCs, based on the response to the direct question along with low scores on MAC-Q, while other subjects had no SMCs and high MAC-Q scores.^18^ Performance on a memory test did not differ according to the MAC-Q results, mirroring our findings, although was worse in those individuals presenting SMCs upon direct questioning.

High age, female gender and low education have all been associated with an increased prevalence of SMCs. Our sample is composed exclusively by elderly subjects (≥60 years), with a predominance of women (39 vs. 21) and with low to middle educational level, especially comparing with studies conducted in North America, Europe or Japan. These features might explain the high percentage (55%) of individuals presenting SMCs.

Memory complaints are recognized to be frequent within the elderly population, especially among those with depressive and anxiety symptoms.^19,20^ In our study, depression is very unlikely as an explanation for the high prevalence of SMCs, since the presence of significant depressive symptoms, based on the results from the Cornell scale, was part of the exclusion criteria.

SMCs have been more related to future cognitive decline rather than to current memory deficits.^2^ Indeed, in a recent study, the presence of SMCs was found to be a significant predictor of subsequent decline, although without a “dose-effect” relationship.^21^ Moreover, in this same study the investigators observed that the occurrence of SMCs also increased the probability of an unstable diagnosis. We still have no longitudinal data on the cognitive performance of our study participants to be able to contribute to this debate, but we may be able to re-evaluate at least part of the sample in the future in order to replicate such observations.

An issue that has not been addressed in our study is the nature or the type of SMC. It is possible that complaints of memory loss in certain specific situations may be more related than others to objective memory impairment or to the initial phases of a dementing process.^22^ Considering the social and cultural characteristics of the Brazilian population, it is possible that the report of memory deficits of a particular nature or in specific situations, less prone to be influenced by inter-individual variability in judgment, may prove to be a useful indicator of actual cognitive performance.

In conclusion, the presence of SMCs was not associated to objective memory impairment or to other cognitive deficits in this group of elderly subjects. Inclusion of additional individuals, with more advanced ages, and also the collection of longitudinal data on their cognitive performance over time is warranted, so as to further investigate the relevance of these symptoms in the Brazilian aged population.
